# Digital Solutions to Optimize Guideline-Directed Medical Therapy Prescriptions in Heart Failure Patients: Current Applications and Future Directions

**DOI:** 10.1007/s11897-024-00649-x

**Published:** 2024-02-16

**Authors:** Jelle P. Man, Joanna Klopotowska, Folkert W. Asselbergs, M. Louis Handoko, Steven A. J. Chamuleau, Mark J. Schuuring

**Affiliations:** 1 Department of Cardiology, Amsterdam University Medical Center, Meibergdreef 9, 1105 AZ Amsterdam, The Netherlands; 2 Netherlands Heart Institute, Utrecht, The Netherlands; 3 Department of Medical Informatics, Amsterdam University Medical Center, Amsterdam, The Netherlands; 4 Amsterdam Public Health, Amsterdam, The Netherlands; 5 Institute of Health Informatics, University College London, London, UK; 6 The National Institute for Health Research University College London Hospitals Biomedical Research Centre, University College London, London, UK; 7 Amsterdam Cardiovascular Sciences, Amsterdam University Medical Centre, University of Amsterdam, Amsterdam, The Netherlands

**Keywords:** Digital health, Digital solutions, Heart failure, Guideline recommendations, GDMT, eHealth

## Abstract

**Purposeof Review:**

Guideline-directed medical therapy (GDMT) underuse is common in heart failure (HF) patients. Digital solutions have the potential to support medical professionals to optimize GDMT prescriptions in a growing HF population. We aimed to review current literature on the effectiveness of digital solutions on optimization of GDMT prescriptions in patients with HF.

**Recent Findings:**

We report on the efficacy, characteristics of the study, and population of published digital solutions for GDMT optimization. The following digital solutions are discussed: teleconsultation, telemonitoring, cardiac implantable electronic devices, clinical decision support embedded within electronic health records, and multifaceted interventions. Effect of digital solutions is reported in dedicated studies, retrospective studies, or larger studies with another focus that also commented on GDMT use. Overall, we see more studies on digital solutions that report a significant increase in GDMT use. However, there is a large heterogeneity in study design, outcomes used, and populations studied, which hampers comparison of the different digital solutions. Barriers, facilitators, study designs, and future directions are discussed.

**Summary:**

There remains a need for well-designed evaluation studies to determine safety and effectiveness of digital solutions for GDMT optimization in patients with HF. Based on this review, measuring and controlling vital signs in telemedicine studies should be encouraged, professionals should be actively alerted about suboptimal GDMT, the researchers should consider employing multifaceted digital solutions to optimize effectiveness, and use study designs that fit the unique sociotechnical aspects of digital solutions. Future directions are expected to include artificial intelligence solutions to handle larger datasets and relieve medical professional’s workload.

## Introduction

There is an epidemic growth in the amount of heart failure (HF) patients and a further increase in the number of patients with HF is projected. Morbidity and mortality of HF patients remain high despite advances in medical therapy in the last decades [1–3]. Current 2021 European Society of Cardiology (ESC) Guidelines for the diagnosis and treatment of acute and chronic HF and the 2023 Focused Update include clear recommendations about pharmacotherapy in patients with HF with a reduced ejection fraction (HFrEF) [1, 4]. These recommendations include the prescription of angiotensin-receptor neprilysine-inhibitors (ARNI) or ACE inhibitors, sodium-glucose cotransporter-2 inhibitors (SGLT2-i), mineralocorticoid receptor antagonists (MRA), and β-blockers, also known as guideline-directed medical therapy (GDMT). The estimated aggregate benefit for HFrEF is greatest for a combination of those foundational four medication classes, defined as guideline-directed medical therapy (GDMT) [5, 6]. Nowadays, it is advised to initiate GDMT with rapid sequencing [7–11]. However, in practice there is a high proportion of slow optimization, low target dose achievement, and/or discontinuation of GDMT [12, 13].

**Fig. 1:**
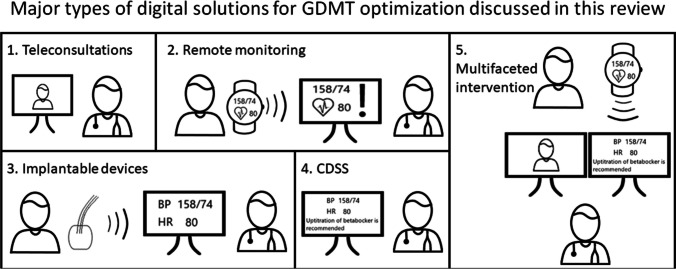
Major types of digital solutions for GDMT optimization discussed in this review

Digital solutions are increasingly used in clinical practice and have the potential to keep healthcare sustainable [14–18]. Digital health refers to the use of information and communications technologies in medicine and other health professions to manage illnesses and health risks and to promote wellness. Digital health has a broad scope and includes the use of wearable devices, mobile health, telehealth, health information technology, and telemedicine [19–21]. Five major digital solutions for GDMT optimization are discussed in this review: 1) teleconsultation (provider to provider and provider to patient), 2) telemonitoring, 3) cardiac implantable electronic devices, 4) clinical decision support systems (CDSS), and 5) multifaceted interventions. These categories are illustrated in Fig. 1.

In this narrative review, we provide in-depth discussion per digital solution within the context of GDMT in patients with HF. Our objective is to provide evidence-based advice about which strategies are the most promising, by distinguishing between successful, as well as less successful digital solutions. Additionally, we explore the potential and future perspectives of leveraging artificial intelligence (AI) as a transformative tool in optimizing GDMT for HF patients, shedding light on how AI can revolutionize and enhance patient outcomes in the realm of GDMT optimization.

## Method

The Medline (Ovid) database was searched in collaboration with an expert librarian using Heart failure, Telemedicine, Decision Support Systems, Electronic Health Records, Monitoring, and guideline directed medical therapy as keywords. ASReview, a semi-automatic machine learning tool for systematic reviews, was subsequently used for screening for relevant articles [22–24]. Articles from 1946 until 2023 were screened using ASReview. From the total output of the Medline database, the first 268 articles were screened for relevance and known relevant articles were marked in the database. Experts in the field of heart failure and digital solutions added any missing articles not included in this list. Based on the marked relevant articles, a ranking is generated by ASReview. This ranking was screened up until 25 irrelevant articles were consecutively encountered [23, 24]. After 25 consecutively encountered irrelevant articles, it is namely less likely that articles lower in the ranking will be relevant. This resulted in 32 articles that used digital solutions for GDMT optimization. The articles are listed per category in Table 1.

**Table 1:** Studies on digital solutions to optimize guideline directed medical therapy in heart failure patients

	First author (acronym)	Year	Design	Sites	Population	*n* (total)	*n* (int)	FU (m)	Brief description of intervention	Effect of intervention
Teleconsultation (peer to peer)										
	Bhatt (IMPLEMENT-HF)	2021	Prospective cohort (pilot)	1	Hospitalized HFrEF	118	89	1	Assess the safety and effectiveness of a virtual care team containing cardiologists and pharmacists for guiding GDMT optimization (pilot study)	Increase in β-blocker, ARNI, MRA, and triple therapy
	Bhatt	2023	Prospective cohort	3	Hospitalized HFrEF	252	83	6	Assess the safety and effectiveness of a virtual care team containing cardiologists and pharmacists for guiding GDMT optimization	Increase GDMT optimization score
	Rao	2023	RCT	1	Non-cardiology ward patients	91	52	1	Investigate the efficacy of a multidisciplinary virtual peer-to-peer HF consult intervention designed to increase GDMT use among patients hospitalized to noncardiology medical services	Increase proportion of patients on ACE/ARB/ARNI
	Wakefield	2009	RCT	1	Hospitalized HF	148	52	3		More likely medication adjustments
Teleconsultation (provider to patient)										
	Sammour	2022	Retrospective cohort	?	HFrEF	5439	2610	?	Outpatient management of guideline-directed medical therapy for heart failure using telehealth	MRA and SGLT2i more often started than telephone contact but equal to in-office
	Wakefield	2009	RCT	1	Hospitalized HF	148	52	3	Evaluate the efficacy of a videophone application used by nurses to reach-out to HF patients	More likely medication adjustments
	Yuan	2021	Retrospective cohort	31	Ambulatory HF during COVID	10,591	1009	9	Investigation of clinical practice patterns of remote cardiology visits during the COVID-19 pandemic	Lower β-blocker, ACEi/ARB/ARNI, and MRA as compared to in person visit
Telemonitoring										
	Antonicelli	2008	RCT (pilot)	1	Congestive HFrEF	57	28	12	Telemonitoring at home in the management of elderly patients with congestive HFrEF	Increase β-blocker use
	Artanian	2020	RCT (pilot)	1	Stable HFrEF	42	21	6	Telemonitoring using monitor daily weight, blood pressure, heart rate, and symptoms	Higher proportion of optimal GDMT doses, decreased time to dose optimization
	Brahmbhatt	2022	Retrospective cohort	5	HF	108	56	24	Telemonitoring platform using patient-reported symptoms and trends in heart rate, blood pressure, and weight	Increase number of patients achieving maximum dose, and earlier
	Dierckx	2015	Retrospective cohort	?	HFrEF	333	278	6	Telemonitoring using measurements of weight, heart rate, and blood pressure and patient reported symptoms	Similar β-blocker, ACE-I/ARB, and MRA use
	Giordano	2011	Retrospective cohort	1	Chronic HFrEF	358	238	6	Telemonitoring at home based on heart rate, blood pressure, 1 lead EKG, and weight	Increase in β-blocker
	Koehler (TIM-HF2)	2018	RCT	113	LVEF < 45% NYHA II/III	1571	796	12	Telemonitoring using both BP monitors and electronic weight scales	3546 medication (likely GDMT) changes
	McLachlan	2021	Prospective cohort	1	Acute HF and HFrEF	50	50	2	Telemonitoring using BP monitors and electronic scales and telephone support	Increase use renin angiotensin blocker, beta-blocker, spironolacton, and ARNI
	Romero	2023	RCT (pilot)	1	HFrEF	55		6	Telemonitoring using wireless devices to transmit heart rate, blood pressure, and weight data	Increase GDMT optimization score
	Samsky (VITAL-HF)	2023	Prospective cohort	1	HFrEF	12	12	3	Telemonitoring using BP, heart rate, and patient reported symptoms	10 initiations, 52 up-, and 13 down-titration GDMT
	Wong (DAVID-HF)	2022	Prospective cohort	1	HFrEF	20	20	4	Telemonitoring using a monitor capable of tracking heart rate, heart rate variability, blood pulse wave, oxygen saturation, respiration rate, skin temperature, electrodermal activity, and steps count	Increase GDMT target dose
Cardiac implantable electronic devices										
	Adamson (CHAMPION)	2016	RCT post-hoc analysis	64	NYHA III, previous HF admission	550	245	6	Remote monitoring of pulmonary artery pressure	More optimization ACEi, β-blocker, and loop diuretic
	Brugts (MONITOR-HF)	2023	RCT	25	Chronic HF NYHA III	348	176	48	Remote monitoring of pulmonary artery pressure	Individualized modification of GDMT
	D’Onofrio	2015	Post-hoc	25	ICD/CRTD patients	987	499	12	Remote ICD monitoring consisting of rhythm and device related measurements	No association RM and β-blocker use
	Hernandez (MANAGE-HF)	2022	Prospective cohort	29	HFrEF and CIED	200	200	12	Algorithm based remote monitoring including measurements of heart sounds, respiration, thoracic impedance and heart rate	GDMT were increased during 74% of the alert cases
	Zile (GUIDE-HF)	2021	RCT	118	Chronic HF NYHA II–IV	1022	497	12	Remote monitoring of pulmonary artery pressure	
EHR support										
	Ahmad (REVEAL-HF)	2022	RCT	4	Hospitalized HF	3124	1590	12	Alerts sent to clinicians on 1-year mortality risk	No effect on GDMT prescription rate
	Allen (EPIC-HF)	2021	RCT	6	HFrEF	290	145	1	Electronically delivered patient activation tool to activate patients with regards to GDMT optimalization	Intensification of GDMT
	Ghazi (PROMPT-HF)	2022	Cluster RCT	4	Ambulatory HFrEF	1310	1310	1	alerts sent to clinicians regarding GDMT optimalization in an outpatient setting	Higher rates of GDMT
	Ghazi (PROMPT-AHF)	2023	Cluster RCT	4	Hospitalized HF	1012	1012	*	Alerts sent to clinicians regarding GDMT optimalization during HF hospitalization	No higher rates of GDMT
	McCarren	2013	Cluster RCT	12	All HF patients	220	220	6	A report containing a list of patients not meeting GDMT goals of β-blockers therapy was sent to healthcare professionals	Increase in β-blocker
	Mukhopadhyay (BETTER CARE-HF)	2023	Cluster RCT	?	HFrEF	2211	755	1	Sending automated, patient-specific, electronic health record-embedded alerts on MRA prescription	Increased MRA use
Multifaceted interventions										
	Gulizia (BLITZ-HF)	2022	Cross-sectional	106	Acute and chronic HF	7218	7218	3	A web-based recording system with alerts on GDMT was used interspersed by face-to-face macro-regional benchmark analyses and educational meetings for clinicians	Ambulatory HFrEF patients increase in ARNI
	Lynch	2022	Prospective cohort	1	Any HF	38	38	12	Integration of remote monitoring using vital signs, questionnaires regarding general health, and pharmacist consultations into an HF-specific disease management program	Increased number and dose GDMT
	Rahimi (SUPPORT-HF2)	2020	RCT	7	HFrEF	202	101	6	On top of telemonitoring (in both intervention and control group) the patients in the intervention group received additional regular feedback to support self-management and the primary care doctors received instructions on blood investigations and pharmacological treatment	No improvement GDMT
	Slade	2022	Prospective cohort	1	HFrEF	12	12	14	A pharmacist-led titration clinic with a standardized titration protocol, and a patient dashboard to identify actionable patients	ACEI/ARB/ARNI/beta-blocker at ≥ 50% target doses increased
	Verma (DASH-HF)	2023	RCT	1	HFrEF	300	150	3	EMR-based HF dashboard (including hospitalization risk scores, VA hospitalizations in the past 12 months, vital signs, laboratory values, active GDMT prescriptions, and upcoming appointments) and a structured telehealth program	No improvement GDMT optimization score

*HER*, electronic health records; *FU*, follow-up, *GDMT*, guideline-directed medical therapy; *HF*, heart failure; *RM*, remote monitoring; *HFrEF*, heart failure reduced ejection fraction; *n*, number; *m*, months; *RCT*, randomized controlled trial; *ARNI*, angiotensin receptor neprilysine-inhibitor; *MRA*, mineralocorticoid receptor antagonist; * variable (discharge)

### Teleconsultation Provider to Provider

Teleconsultation refers to the remote exchange of medical information and advice between a professional and a patient or provider using telecommunication technologies, allowing assessment, monitoring, and management of the condition without an in-person visit [25].

Bhatt et al. reported on results of the IMPLEMENT-HF study in 2021 and 2023 [26, 27]. The authors assessed the safety and effectiveness of a virtual care team containing cardiologists and pharmacists guiding the GDMT optimization of patients hospitalized in non-cardiac departments. Virtual care teams represent a centralized and scalable approach to optimize GDMT. In this multicenter, prospective cohort study, the investigators allocated 252 hospital encounters in patients with HFrEF to a virtual care team-guided strategy or usual care. The virtual care team strategy significantly improved GDMT scores vs. usual care (adjusted difference: 1.2; 95% CI: 0.7–1.8; *P* < 0.001). New initiations (44% vs. 23%; absolute difference: 21%; *P* = 0.001) and net intensifications (44% vs. 24%; absolute difference = 20%; *P* = 0.002) during hospitalization were higher in the virtual care team group, translating to a number needed to intervene of five encounters. Hypotension was the most common safety event, occurring in 17% encounters allocated to usual care and 11% allocated to the virtual care team guided intervention (*P* = 0.28). Rates of acute kidney injury, bradycardia, and hyperkalemia were similar in those allocated to usual care and to the virtual care team-guided intervention. The authors concluded that among patients hospitalized with HFrEF, a virtual care team-guided strategy for GDMT optimization was safe and improved GDMT across multiple hospitals in an integrated health system. Rao et al. also reported on in-hospital virtual provider-to-provider consultations in 91 patients admitted to non-cardiology departments. The virtual care team consisted of cardiologists and pharmacists and the consultation was directed to clinicians of non-cardiology departments. [28]. In this single-center randomized controlled trial (RCT), an increase in the proportion of patients on angiotensin-converting enzyme inhibitors (ACE-I)/angiotensin receptor blockers (ARB)/ARNI was demonstrated.

### Teleconsultation Provider to Patient

Sammour et al. reported on outpatient management of GDMT for patients with HFrEF using video contact [29]. This retrospective cohort study included 5439 patients with HFrEF. The authors concluded that the initiation of GDMT for HFrEF was similar between in-office and video visits but lower with telephone visits, whereas the initiation of a loop diuretic was less frequent in both types of remote visits. In RCT by Wakefield et al. the efficacy of a videophone application used by nurses to reach-out to HF patients each week for 90 days after hospital discharge was evaluated [30]. A total of 148 patients were enrolled: 49 were randomized to usual care, 52 to the videophone intervention, and 47 to the telephone intervention. The videophone intervention group was more likely to have GDMT medications adjusted during the 90-day intervention period in comparison to telephone intervention and usual care patients. Yuan et al. investigated clinical practice patterns of remote cardiology visits during the COVID-19 pandemic. This study reported lower use of GDMT in the teleconsultation arm as compared to standard care [31]. The authors included in their retrospective cohort study all outpatient cardiology visits for HF at a multisite healthcare system during the COVID pandemic. During remote visits, medical professionals were less likely to order diagnostic testing (odds ratio, 0.20 [0.18–0.22] video versus in-person, 0.18 [0.17–0.19] telephone versus in-person) or prescribe β-blockers (0.82 [0.68–0.99], 0.35 [0.26–0.47]), MRA (0.69 [0.50–0.96], 0.48 [0.35–0.66]), or loop diuretics (0.67 [0.53–0.85], 0.45 [0.37–0.55]). During telephone visits, medical professionals were less likely to prescribe ACE-I/ARB /ARNIs; 0.54 [0.40–0.72]). The authors concluded that remote visits for HFrEF care were associated with reduced diagnostic testing and GDMT. The reduced diagnostic testing may be partially explained by the following factors associated with COVID-19 pandemic: a reluctance of clinicians who preferred remote visits during the pandemic to send the patient to the hospital, a reluctance of patients to go to the hospital for a diagnostic test or blood test, and an underdeveloped infrastructure while using teleconsultations regarding the ordering of blood tests, medicine recipes, and diagnostic tests.

## Telemonitoring

Telemonitoring involves the monitoring of a patient’s vital signs, symptoms, or health data using technology such as wearable devices, sensors, or digital platforms. The collected information is transmitted to medical professionals for assessment, enabling proactive healthcare management and interventions. Of the 10 studies on telemonitoring to optimize GDMT, 9 (90%) report positive results. In a single-center RCT, Antonicelli et al. studied the impact of telemonitoring at home on the management of elderly patients with congestive HFrEF [32]. Fifty-seven congestive patients with HF were randomized to standard care or to home telemonitoring-based care and followed for 12 months. In the patients who were telemonitored, weekly reports on their clinical status were obtained and their management was modified accordingly. Home telemonitoring was associated with more frequent use of β-blockers. Artanian et al. conducted a pilot RCT on the impact of remote dose titration combined with telemonitoring on the GDMT optimization for patients with HFrEF [33, 34]. A total of 42 patients with new-onset (10/42, 24%) and existing (32/42, 76%) HFrEF were randomized. Within 6 months of enrollment, 86% (18/21) of patients in the telemonitoring group achieved optimal doses versus 48% (10/21) of patients in the control group. The median time to dose optimization was 11.0 weeks for the telemonitoring group versus 18.8 weeks for the control group. Brahmbhatt et al. reported on a multicenter RCT in 108 cardiac outpatients with a diagnosis of HFrEF [35, 36]. Here, a non-invasive telemonitoring platform was used to allow daily nurse coordinator-led assessment of trends in heart rate, blood pressure (BP), and weight. The telemonitoring data were used to make decisions on optimization of GDMT every 2 weeks. This intervention resulted in more patients achieving maximum tolerated doses, and on average 2 months earlier. In a retrospective cohort study by Giordano et al., an increase in β-blockers use during an 8-year period in 358 patients with chronic HFrEF was found [37]. During a 6-month home-based telemonitoring program, there was a significant increase in the mean daily dosage of β-blockers prescribed. Samsky et al. reported in the VITAL-HF cohort study on the efficacy and patient perspectives of the Story Health web-based platform in 12 HFrEF patients [38]. Automated alerts were triggered based on pre-specified vital signs and laboratory data. GDMT optimization plans were individually created in the digital platform by local medical professionals. There were 10 GDMT initiations, 52 up-titrations, and 13 down-titrations. They also reported that the intervention alleviated concerns associated with the uncertainty in daily living, led to an increased feeling of security, and empowered patients to understand decision-making regarding GDMT. A larger study is ongoing (NCT05602454). Koehler et al. published in 2018 the results of the TIM-HF2 RCT [39]. This multicenter study enrolled 1571 HF patients with left ventricular ejection fraction < 45% and NYHA II or III. In the telemonitoring group, medication changes were often performed (*n* = 3546). McLachlan et al. studied, in a prospective cohort study, 50 consecutive HFrEF patients using both BP monitors and electronic weight scales and remote nurse practitioner support during the COVID pandemic [40]. The authors reported rapid dose titration with less need for clinic review with optimization rates comparable with most usual care. In a RCT by Romero et al., 55 patients with HFrEF who were randomly assigned to receive either usual care or a usual care and quality-improvement remote dose titration with telemonitoring intervention [41]. The intervention group used wireless devices to transmit heart rate, BP, and weight data daily, which were remotely reviewed by cardiologists and nurses every 2–4 weeks. At the 6-month follow-up, the intervention group had a GDMT score (a comparison of the used dose to the target dose of each medication) of 64.6% compared to 56.5% in the control group (*p* = 0.01). Wong et al. conducted the DAVID-HF prospective cohort study on wearable armband monitors paired with a smartphone application in 20 HFrEF patients [42]. A medication optimization algorithm was used to adjust medication daily. At 120 days, 70% received ≥ 50% maximal target dose ACE-I/ARB/ARNI (*P* = 0.110) with percent maximal target dose increased to 64.4 + 33.5% (*P* = 0.060). The proportion receiving ≥ 50% maximal target dose ARNI increased from 15 to 55% (*P* = 0.089) with % maximal target dose ARNI increased from 20.6 + 30.9 to 53.1 + 39.5% (*P* = 0.006). More patients received ≥ 50% maximal target dose MRA (65 vs. 25%, *P* = 0.011) with % mean target dose MRA increased from 25.0 + 19.9 to 46.2 + 28.8% (*P* = 0.009).

Neutral study results have been reported by Dierickx et al. where the impact of home telemonitoring supported by a nurse-specialist in a “real-world” setting was studied in a retrospective cohort [43]. The authors analyzed data on 333 patients with HFrEF. After 6 months, prescription of β-blockers (92% vs. 83%), ACE-I/ARB (92% vs. 90%) and MRA (68% vs. 67%) did not differ significantly between the home telemonitoring and usual care group. The proportions of patients who achieved ≥ 50% and ≥ 100% of target doses of β-blockers, ACE-I/ARB, and MRA were also similar in each group.

## Cardiac Implantable Electronic Devices

Cardiac implantable electronic devices (CIED) are devices placed to perform invasive telemonitoring and manage cardiac status. Specific devices include pulmonary artery pressure (PAP) monitors not only to assist in HF management and pacemakers or implantable cardioverter-defibrillators (ICD) to help regulate arrhythmia but also provide data for remote HF management. Of the four studies on CIED, three (75%) showed positive results on GDMT optimization. Results from the CHAMPION RCT post-hos analysis by Adamson et al. on PAP monitors where pressures were remotely made available to investigators demonstrated that GDMT was changed more often in the remote group using pressure information compared with the control group using symptoms and daily weights alone [44, 45]. Brugts et al. reported in the MONITOR-HF RCT on the effect of remote PAP monitoring on the quality of life [46]. Individualized optimization of GDMT was found during 48 months of follow-up. Zile et al. also reported on the effectiveness of remote PAP monitoring in the GUIDE-HF trial and included a total of 200 patients with HFrEF and New York heart association (NYHA) class II/III. In this RCT, 70% more medication changes occurred in the treatment group compared to the control group (*P* < 0.001). This study however only reported on the total amount of medication changes and stated that diuretic changes were the most frequent compared to the four foundational medication groups for HFrEF. Hernandez et al. performed the MANAGE-HF prospective cohort study in 2022 [47] and included a total of 200 patients with HFrEF, NYHA class II/III, who received a cardiac resynchronization therapy-defibrillator or ICD in combination with remote data monitoring who had either a hospitalization for HF or unscheduled visit for HF exacerbation or an elevated natriuretic peptide. GDMT was optimized during 74% of the alert of decongestion.

However, post-hoc analysis by D’Onofrio et al. where the impact of ICD with remote ICD data monitoring was investigated on the dose of β-blockers achieved, and its association with clinical outcome at 12 month showed negative results [48]. Altogether, 987 consecutive patients were enrolled and followed up for at least 12 months in 25 Italian centers. Telemonitoring comprising interrogation and transmission of ICD data through a connection at scheduled intervals or in the case of programmable alert conditions, without patient intervention, was adopted by 499 patients. The number of patients receiving β-blockers at any dose decreased after 12 months (from 403 (81%) to 370 (74%) for the remote arm and from 389 (80%) to 342 (70%) for the standard arm, both *p* < 0.02). Nonetheless, the number of patients on β-blockers at the effective dose increased in both arms (from 60 (12%) to 82 (16%) for remote and from 63 (13%) to 98 (20%) for standard arms, respectively, both *p* < 0.05). In a multivariate analysis, remote ICD data monitoring was not associated with an effective dose of β-blockers at the follow-up evaluation. The authors concluded that in a “real-world” setting, there was no association between remote ICD data monitoring and the achieved dose of β-blockers. For data from the pre-COVID-19 period, there were 70% more medication changes in the treatment group compared with the control group with 1.19 changes/patient-month in treatment vs. 0.700 changes/patient-month in control (*P* < 0.001).

## Clinical Decision Support Systems

CDSS can nudge the clinician to adhere to disease-specific guidelines via among others alerts, reports, and e-messages, to influence medical professionals’ decision-making by presenting information in a way that encourages optimal clinical practices, such as optimizing GDMT. Of the six studies on CDSS to optimize GDMT, four (67%) showed positive results. Allen et al. reported the results of EPIC-HF RCT [49]. This study randomized patients with HFrEF to usual care versus patient activation tools—a 3-min video and 1-page checklist—delivered electronically 1 week before, 3 days before, and 24 h before a cardiology clinic visit. The EPIC-HF enrolled 306 patients, 290 of whom attended a clinic visit during the study period: 145 were sent the patient activation tools and 145 were controls. The authors reported that this patient activation tool delivered electronically before a cardiology clinic visit improved medical professionals’ optimization of GDMT. An explanation given for the effectiveness of this method is that the tools engaged and activated patients before the clinical encounter. The PROMPT-HF cluster RCT by Ghazi et al. focused on outpatient CDSS alerts to improve GDMT [50, 51]. The study enrolled 1310 outpatient patients with HFrEF. The primary outcome of increase in number of prescribed GDMT at 30 days occurred in 176 of 685 (26%) participants in the alert arm vs. 117 of 625 (19%) in the usual care arm, thus increasing GDMT use by > 40% after alert exposure (adjusted relative risk: 1.41; 95% CI: 1.03–1.93; *P = *0.03). The number of patients needed to alert to result in GDMT increase was 14. A total of 79% of alerted professionals agreed that the alert was effective at enabling improved GDMT. In a cluster randomized trial by McCarren et al., a CDSS system was used to evaluate its effectiveness on β-blocker uptitration [52]. The trial included 220 patients among 12 centers. A report containing a list of patients not meeting GDMT goals of β-blockers therapy was sent to healthcare professionals for 6 months. The CDSS was associated with 1.9-fold greater odds of improvement in prescribing and a greater odd of a higher dose (1.9, 95% CI 1.1–3.3) of GDMT. In the BETTER CARE-HF cluster RCT, Mukhodadhyay et al. reported on the effect of outpatient CDSS alerts and messages to improve MRA prescriptions [53]. The study included 2211 patients (alert: 755, electronic health record (EHR) messages: 812, usual care [control]: 644). The alert more than doubled MRA prescribing compared to usual care (relative risk: 2.53; 95% CI: 1.77–3.62; *P* < 0.0001) and improved MRA prescribing compared to the message (relative risk: 1.67; 95% CI: 1.21–2.29; *P* < 0.002). The number of patients with alert needed to result in an additional MRA prescription was 5.6.

Interestingly, the second PROMPT cluster RCT, PROMPT-AHF also by Ghazi et al. in hospitalized patients, showed negative study results [54]. In total 1012 patients were enrolled. The CDSS included a best practice advice that was displayed to professionals upon accessing a patient’s EHR and engaging with the order entry interface. The primary outcome was an increase in the number of GDMT prescriptions at discharge, and this occurred in 34% of both the alert and no alert groups (*P* = 0.99]. Patients were randomized to either the control arm or the alert arm. Patients in the alert arm were more likely to have an increase in MRA [adjusted RR: 1.54 (1.10, 2.16), *P* = 0.01]. Reasons given for the negative results are a focus on multiple other comorbidities, a pressure to achieve a fast discharge, and alert fatigue due to multiple other in-hospital alerts. In the REVEAL-HF RCT, Ahmad et al. evaluated CDSS alerts including the 1-year mortality calculated using an algorithm that was derived and validated using similar historic patients in the EHR [55]. GDMT prescription rates were recorded and remained comparable at discharge. Reasons given for the ineffectiveness were that no direct treatment advice was given in the alert and that clinicians could have an aversion to algorithm-derived prognoses.

## Multifaceted Interventions

In five studies several digital and non-digital interventions were combined to a multifaceted approach [56]. Here, three studies (60%) showed positive results. Guliza et al. performed the BLITZ-HF cross-sectional study [57]. In the BLITZ-HF study, a web-based recording system with alerts on GDMT was used for two 3-month enrolment periods carried out 3 months apart, interspersed by face-to-face macro-regional benchmark analyses and educational meetings for clinicians. In total 7218 patients with acute and chronic HF were enrolled at 106 cardiology sites. A significant increase in ARNI prescription rates was observed. Lynch et al. evaluated, in a prospective cohort study, a proactive integration of telemonitoring and remote pharmacist consultations among 16 patients [58]. Use of GDMT increased by 17.1% (*p* < 0.001), the number of patients receiving GDMT increased from 3 to 11 (*p* = 0.008), GDMT dose optimization increased by 25.3% (*p* < 0.001), and the number of patients maximally optimized on GDMT increased from 1 to 6 (*p* = 0.06). In a prospective cohort study by Slade et al., the effectiveness of a pharmacist-led HF medication titration clinic with a standardized titration protocol and a patient dashboard was tested in 12 patients [59]. In 14 months, the prescribing of ACEI/ARB/ARNI and β-blockers therapy at ≥ 50% target doses for patients with HFrEF was increased. This study demonstrated the potential of a multifaceted pharmacist-led approach that integrates population-level interventions such as clinical dashboard management with a HF medication optimization clinic.

Nonetheless, there are also negative studies on multifaceted interventions. Rahimi et al. performed the SUPPORT-HF2 RCT at seven sites in the UK and recruited a total of 202 patients with HFrEF [60]. Patients randomized to the intervention received additional regular feedback via a telephone to support self-management and their primary care doctors received digital instructions on blood investigations and pharmacological treatment. There was no evidence for GDMT improvement. This can be explained by the chosen study design in which the control group also receives extensive telemonitoring and submits questionnaires about their well-being resulting in a diluted intervention effect. Verma et al. performed the DASH-HF RCT in 300 veterans with HFrEF [61]. The intervention was a HF dashboard in the EHR to monitor and improve outpatient HF management. No significant difference was found between the intervention arm and usual care arm in GDMT optimization score. Reasons given for the ineffectiveness of the intervention were a low response rate of telephone contact of the patient after the digital alert and a low treatment effect because of otherwise unsuitable infrastructure apart from the CDSS.

## Discussion

In this review five major digital solutions were discussed. The effect of digital solutions on GDMT is summarized from dedicated studies, retrospective studies, and larger studies with another primary objective that also commented on GDMT use. Overall, we see more positive than negative results. However, there is a large heterogeneity in study design, outcomes used, and populations studied, which hampers comparison of different digital solutions. Furthermore, a substantial number of studies were conducted during the COVID pandemic. Therefore, the generalizability of the results to post-COVID practice is difficult [62, 63].

The path to achieve GDMT optimization is evaluated by a heterogeneity of study designs, showcasing a landscape rich in diversity. A cluster randomized trial at institution level might mitigate the bias resulting from the learning experience of a clinician treating both intervention and control patients. However, cluster randomized controlled trails are resource intensive and may encounter challenges in controlling for confounding variables at the individual level within clustered groups. Moreover, all clusters should be ready for enrollment from baseline which is often a challenge in clinical practice. Stepped wedge RCT can be a solution to this; however, this has the disadvantage of introducing a time bias. Therefore, a traditional RCT design is commonly chosen. A trial design which diminishes the risk of placebo effect might also be chosen, like in the study of Rahimi et al. [60]. In this design some form of telemonitoring is also performed in the control group. If the objective of the study is to measure the effect of regular feedback to support self-management and/or the effect of sending instructions to primary caregivers on top of the performed telemonitoring, this design may be effective to diminish the placebo effect and the feeling of “losing” when a patient is assigned to the less extensive digital intervention group. Such a design might however also dilute the intervention effect of a multifaceted digital intervention as patients in the control arm also need to conduct some form of telemonitoring or at home measurements. Use of GDMT can also be extracted from retrospective studies or larger studies with another focus; however, conclusions should be drawn with caution due to the large risk of biases. Furthermore, a more comprehensive understanding of the underlying problems related to low GDMT use and slow up titration is needed to deliver more effective digital solutions [64]. Therefore, interviewing patients and clinicians may be useful to better understand the underlying problems and improve the intervention, but this may be less suitable for large patient populations containing different local cultures [65]. Regarding the type of intervention, a multifaceted solution where multiple interventions are combined seems to be a more appealing strategy than solely one digital solution [66]**. **A multifaceted approach can help to achieve a synergistic effect to aim for the most optimal results at patient and provider levels. Also, interventions on a specific target, for instance, on a drug (group) that is the most underused, might be more impactful than a broad intervention on all four drug groups included in the GDMT.

It is important to identify barriers and facilitators when implementing digital solution for GDMT optimization. Reimbursement, costs, and resistance to change are known barriers for digital solutions, as well as a lack of integration of the solutions in EHR systems [67]. Academic institutions that conduct research, entrepreneurs and startups, patient advocacy groups, and insurance and payer organizations are known facilitators for digital initiatives. In the included studies**,** some authors elaborated on barriers and facilitators. Facilitators included the technical opportunities and the COVID-19 pandemic. The barriers included counseling of non-English-speaking patients on multiple medication changes, accessing and using required technology, decreased clarity of communication, an inability to perform comprehensive physical examinations, cost-related barriers, knowledge of and comfort with the drug therapy optimization, passive recommendations rather than active, patient preferences, and alert fatigue. Prioritizing education and training for users to enhance technology literacy, an implementation of solutions that are a replacement of usual care and not an additional workload, establishing robust data privacy and security measures to build trust, are ways to overcome some of these barriers hampering the adoption of digital solutions to optimize GDMT.

## Future Directions

We foresee more innovation in the field of digital solutions in cardiology in the near future [68, 69]. Future digital solutions to optimize GDMT are expected to include AI-based technology to handle larger datasets and reduce workload of healthcare professionals [66, 67]**.** We identified two studies including AI-based technology that are ongoing and registered on www.clinicaltrials.gov. NCT04394754 is a RCT evaluating the efficacy of AI-based technology in the treatment of congestive HF. A “smart” scale (Bodyport), an automated conversational platform (Conversa), and a coaching application (Noom) using an AI algorithm are used to determine fluid status noninvasively and assess HF risk [70]. In total 182 participants are expected to enroll in this study. The NCT04191330 is a RCT where digital AI-powered algorithms using a cloud-based platform (BiovitalsHF) combined with wearable sensors are used to aid management of optimization of GDMT prescriptions outside of normal or traditional clinical encounters. In total 228 participants are expected to enroll in this study.

**Fig. 2:**
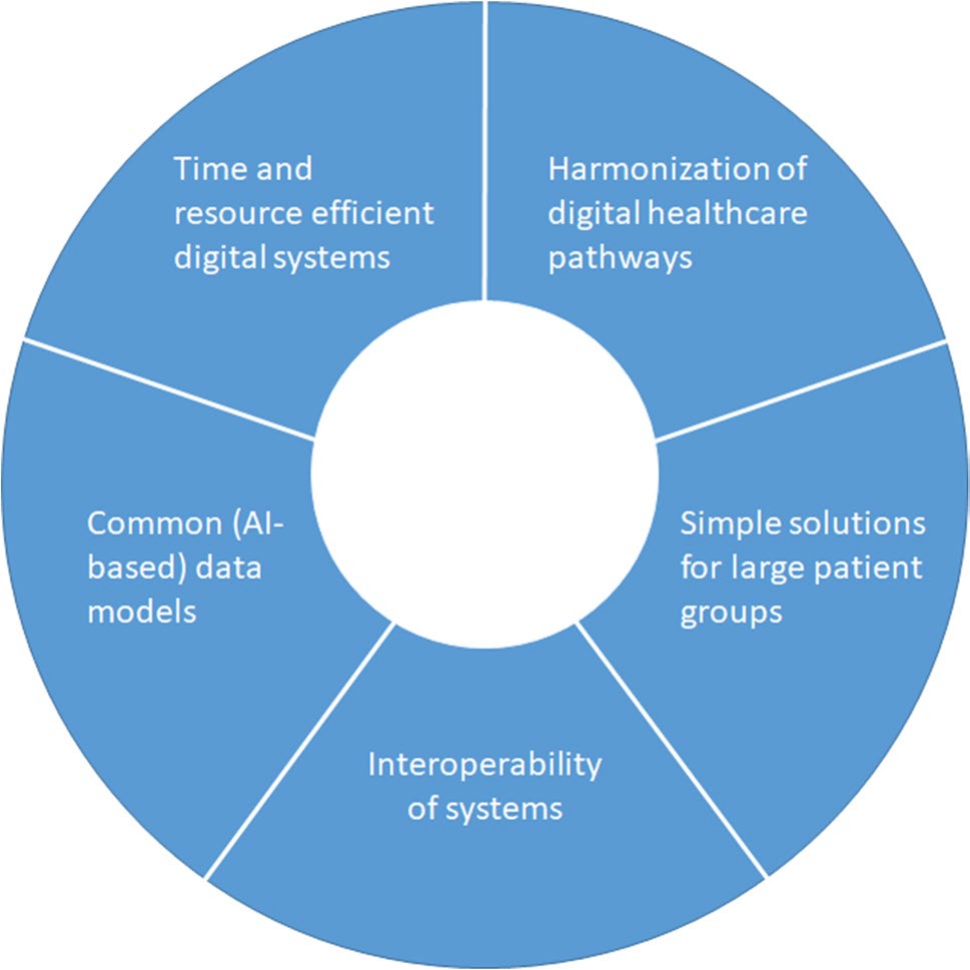
Necessities for a successful implementation of digital solutions for GDMT optimization in multiple hospitals

In our opinion, the deployment of digital systems to optimize GDMT in multiple hospitals and an evaluation of these deployed systems are the next steps to draw more definitive conclusions on the effectiveness of digital solutions for GDMT optimization. As illustrated in Fig. 2, interoperability of systems, harmonization of healthcare pathways, deployment of simple to use systems suitable for large patient groups, common (AI-based) data models, and time and resource efficient systems are likely to be needed for an efficient and effective deployment of such systems among multiple hospitals.

## Conclusion

Based on this review, measuring and controlling vital signs in telemedicine studies should be encouraged. Professionals should be actively alerted about suboptimal GDMT and the researchers should consider employing multifaceted digital solutions to optimize effectiveness. Also, study designs should be used that fit the unique sociotechnical aspects of digital solutions. There remains a need for well-designed evaluation studies to determine safety and effectiveness of digital solutions for GDMT optimization in patients with HF. Future directions are expected to include artificial intelligence solutions to handle larger datasets and relieve medical professional’s workload.

## References

[1] McDonagh TA, Metra M, Adamo M, Gardner RS, Baumbach A, Böhm M (2021). 2021 ESC Guidelines for the diagnosis and treatment of acute and chronic heart failure: developed by the task force for the diagnosis and treatment of acute and chronic heart failure of the European Society of Cardiology (ESC) With the special contribution of the Heart Failure Association (HFA) of the ESC. Eur Heart J.

[2] Cook C, Cole G, Asaria P, Jabbour R, Francis DP (2014). The annual global economic burden of heart failure. Int J Cardiol.

[3] Hsiao R, Greenberg B (2016). Neprilysin Inhibition as a PARADIGM shift in heart failure therapy. Curr Heart Fail Rep.

[4] McDonagh TA, Metra M, Adamo M, Gardner RS, Baumbach A, Böhm M et al. Focused Update of the 2021 ESC Guidelines for the diagnosis and treatment of acute and chronic heart failure: Developed by the task force for the diagnosis and treatment of acute and chronic heart failure of the European Society of Cardiology (ESC) With the special contribution of the Heart Failure Association (HFA) of the ESC. Eur Heart J. 2023;ehad195.

[5] Tromp J, Ouwerkerk W, van Veldhuisen DJ, Hillege HL, Richards AM, van der Meer P (2022). A systematic review and network meta-analysis of pharmacological treatment of heart failure with reduced ejection fraction. JACC Heart Fail.

[6] Xiang B, Yu Z, Zhou X (2021). Comparative efficacy of medical treatments for chronic heart failure: a network meta-analysis. Front Cardiovasc Med.

[7] Packer M, McMurray JJV (2021). Rapid evidence-based sequencing of foundational drugs for heart failure and a reduced ejection fraction. Eur J Heart Fail.

[8] Mebazaa A, Davison B, Chioncel O, Cohen-Solal A, Diaz R, Filippatos G (2022). Safety, tolerability and efficacy of up-titration of guideline-directed medical therapies for acute heart failure (STRONG-HF): a multinational, open-label, randomised, trial. Lancet.

[9] Sharma A, Verma S, Bhatt DL, Connelly KA, Swiggum E, Vaduganathan M (2022). Optimizing foundational therapies in patients with HFrEF: how do we translate these findings into clinical care?. JACC Basic Transl Sci.

[10] Shen L, Jhund PS, Docherty KF, Vaduganathan M, Petrie MC, Desai AS (2022). Accelerated and personalized therapy for heart failure with reduced ejection fraction. Eur Heart J.

[11] Greene SJ, Butler J, Fonarow GC (2021). Simultaneous or rapid sequence initiation of quadruple medical therapy for heart failure-optimizing therapy with the need for speed. JAMA Cardiol.

[12] Savarese G, Kishi T, Vardeny O, Adamsson Eryd S, Bodegård J, Lund LH, Thuresson M, Bozkurt B. Heart failure drug treatment-inertia, titration, and discontinuation: a multinational observational study (EVOLUTION HF). JACC Heart Fail. 2022;S2213–1779(22)00508-X. 10.1016/j.jchf.2022.08.009 36202739

[13] Brunner-La Rocca H-P, Linssen GC, Smeele FJ, van Drimmelen AA, Schaafsma H-J, Westendorp PH (2019). Contemporary drug treatment of chronic heart failure with reduced ejection fraction: the CHECK-HF registry. JACC Heart Fail.

[14] González-Juanatey JR, Cinza SS (2023). Clinician-to-clinician electronic consultation in cardiology is also a digital health technology for cardiovascular care. Eur Heart J Digit Health.

[15] Jensen MT, Treskes RW, Caiani EG, Casado-Arroyo R, Cowie MR, Dilaveris P (2021). ESC working group on e-cardiology position paper: use of commercially available wearable technology for heart rate and activity tracking in primary and secondary cardiovascular prevention—in collaboration with the European Heart Rhythm Association, European Association of Preventive Cardiology, Association of Cardiovascular Nursing and Allied Professionals, Patient Forum, and the Digital Health Committee. Europ Heart J - Digital Health.

[16] Kauw D, Koole MAC, Winter MM, Dohmen DAJ, Tulevski II, Blok S (2019). Advantages of mobile health in the management of adult patients with congenital heart disease. Int J Med Inform.

[17] Anker SD, Koehler F, Abraham WT (2011). Telemedicine and remote management of patients with heart failure. Lancet.

[18] Cowie MR, Bax J, Bruining N, Cleland JGF, Koehler F, Malik M, Pinto F, van der Velde E, Vardas P (2015). e-Health: a position statement of the European Society of Cardiology. Eur Heart J.

[19] Ahmed S, Kelly YP, Behera TR, Zelen MH, Kuye I, Blakey R (2020). Utility, Appropriateness, and content of electronic consultations across medical subspecialties. Ann Intern Med.

[20] Takahashi EA, Schwamm LH, Adeoye OM, Alabi O, Jahangir E, Misra S (2022). An overview of telehealth in the management of cardiovascular disease: a scientific statement from the American Heart Association. Circulation.

[21] Ronquillo Y, Meyers A, Korvek SJ. Digital Health [Internet]. In: StatPearls. Treasure Island (FL): StatPearls Publishing; 2023 [cited 2023 Oct 10]. Available from: http://www.ncbi.nlm.nih.gov/books/NBK470260/

[22] van Ommen F, Coenen P, Malekzadeh A, de Boer AGEM, Greidanus MA, Duijts SFA (2023). Interventions for work participation of unemployed or work-disabled cancer survivors: a systematic review. Acta Oncol.

[23] Ros R, Bjarnason E, Runeson P. A machine learning approach for semi-automated search and selection in literature studies [Internet]. In: Proceedings of the 21st International Conference on Evaluation and Assessment in Software Engineering. New York, NY, USA: Assoc Comput Mach. 2017;118–127. Available from: 10.1145/3084226.3084243

[24] van de Schoot R, de Bruin J, Schram R, Zahedi P, de Boer J, Weijdema F (2021). An open source machine learning framework for efficient and transparent systematic reviews. Nature Machine Intelligence.

[25] Guasti L, Dilaveris P, Mamas MA, Richter D, Christodorescu R, Lumens J (2022). Digital health in older adults for the prevention and management of cardiovascular diseases and frailty. A clinical consensus statement from the ESC Council for Cardiology Practice/Taskforce on Geriatric Cardiology, the ESC Digital Health Committee and the ESC Working Group on e-Cardiology. ESC Heart Fail..

[26] Bhatt AS, Varshney AS, Nekoui M, Moscone A, Cunningham JW, Jering KS (2021). Virtual optimization of guideline-directed medical therapy in hospitalized patients with heart failure with reduced ejection fraction: the IMPLEMENT-HF pilot study. Eur J Heart Fail.

[27] Bhatt AS, Varshney AS, Moscone A, Claggett BL, Miao ZM, Chatur S (2023). Virtual care team guided management of patients with heart failure during hospitalization. J Am Coll Cardiol.

[28] Rao VN, Shah A, McDermott J, Barnes SG, Murray EM, Kelsey MD (2023). In-hospital virtual peer-to-peer consultation to increase guideline-directed medical therapy for heart failure: a pilot randomized trial. Circ Heart Fail.

[29] Sammour Y, Main ML, Austin BA, Magalski A, Sperry BW (2022). Outpatient management of guideline-directed medical therapy for heart failure using telehealth: a comparison of in-office, video, and telephone visits. J Card Fail.

[30] Wakefield BJ, Holman JE, Ray A, Scherubel M, Burns TL, Kienzle MG (2009). Outcomes of a home telehealth intervention for patients with heart failure. J Telemed Telecare.

[31] Yuan N, Botting PG, Elad Y, Miller SJ, Cheng S, Ebinger JE (2021). Practice patterns and patient outcomes after widespread adoption of remote heart failure care. Circ Heart Fail.

[32] Antonicelli R, Testarmata P, Spazzafumo L, Gagliardi C, Bilo G, Valentini M (2008). Impact of telemonitoring at home on the management of elderly patients with congestive heart failure. J Telemed Telecare.

[33] Artanian V, Ross HJ, Rac VE, O’Sullivan M, Brahmbhatt DH, Seto E (2020). Impact of remote titration combined with telemonitoring on the optimization of guideline-directed medical therapy for patients with heart failure: internal pilot of a randomized controlled trial. JMIR Cardio.

[34] Massot M, Itier R, Galinier M, Roncalli J, Fournier P, Ayot S (2022). Ultra-fast remote up-titration of heart failure treatment: a safe, efficient and feasible protocol. Europ Heart J.

[35] Ware P, Ross HJ, Cafazzo JA, Boodoo C, Munnery M, Seto E (2020). Outcomes of a heart failure telemonitoring program implemented as the standard of care in an outpatient heart function clinic: pretest-posttest pragmatic study. J Med Internet Res.

[36] Brahmbhatt DH, Ross HJ, O Sullivan M, Artanian V, Rac VE, Seto E. Use of a remote telemonitoring platform significantly improves medication optimisation in heart failure patients. Eur Heart J. 2022;43:ehac544.1094.

[37] Giordano A, Zanelli E, Scalvini S (2011). Home-based telemanagement in chronic heart failure: an 8-year single-site experience. J Telemed Telecare.

[38] Samsky MD, Leverty R, Gray JM, Davis A, Fisher B, Govil A (2023). Patient perspectives on digital interventions to manage heart failure medications: the VITAL-HF pilot. J Clin Med.

[39] Koehler F, Koehler K, Deckwart O, Prescher S, Wegscheider K, Kirwan B-A (2018). Efficacy of telemedical interventional management in patients with heart failure (TIM-HF2): a randomised, controlled, parallel-group, unmasked trial. Lancet.

[40] McLachlan A, Aldridge C, Morgan M, Lund M, Gabriel R, Malez V (2021). An NP-led pilot telehealth programme to facilitate guideline-directed medical therapy for heart failure with reduced ejection fraction during the COVID-19 pandemic. N Z Med J.

[41] Romero E, Yala S, Sellers-Porter C, Lynch G, Mwathi V, Hellier Y (2023). Remote monitoring titration clinic to implement guideline-directed therapy for heart failure patients with reduced ejection fraction: a pilot quality-improvement intervention. Front Cardiovasc Med.

[42] Wong CK, Un KC, Zhou M, Cheng Y, Lau YM, Shea PC (2022). Daily ambulatory remote monitoring system for drug escalation in chronic heart failure with reduced ejection fraction: pilot phase of DAVID-HF study. Eur Heart J Digit Health.

[43] Dierckx R, Cleland JG, Pellicori P, Zhang J, Goode K, Putzu P (2015). If home telemonitoring reduces mortality in heart failure, is this just due to better guideline-based treatment?. J Telemed Telecare.

[44] Adamson PB, Abraham WT, Stevenson LW, Desai AS, Lindenfeld J, Bourge RC (2016). Pulmonary artery pressure-guided heart failure management reduces 30-day readmissions. Circ Heart Fail.

[45] Srivastava PK, DeVore AD, Hellkamp AS, Thomas L, Albert NM, Butler J (2021). Heart failure hospitalization and guideline-directed prescribing patterns among heart failure with reduced ejection fraction patients. JACC Heart Fail.

[46] Brugts JJ, Radhoe SP, Clephas PRD, Aydin D, van Gent MWF, Szymanski MK (2023). Remote haemodynamic monitoring of pulmonary artery pressures in patients with chronic heart failure (MONITOR-HF): a randomised clinical trial. Lancet.

[47] Hernandez AF, Albert NM, Allen LA, Ahmed R, Averina V, Boehmer JP (2022). Multiple cArdiac seNsors for mAnaGEment of Heart Failure (MANAGE-HF) - phase I evaluation of the integration and safety of the heartlogic multisensor algorithm in patients with heart failure. J Card Fail.

[48] D’Onofrio A, Stabile G, Capucci A, Amellone C, De Simone A, Leoni L (2016). Association between remote implantable cardioverter defibrillator monitoring and beta-blocker utilization: an analysis from the EFFECT study. J Telemed Telecare.

[49] Allen LA, Venechuk G, McIlvennan CK, Page RL, Knoepke CE, Helmkamp LJ (2021). An electronically delivered patient-activation tool for intensification of medications for chronic heart failure with reduced ejection fraction: the EPIC-HF trial. Circulation.

[50] Ghazi L, Yamamoto Y, Riello RJ, Coronel-Moreno C, Martin M, O’Connor KD (2022). Electronic alerts to improve heart failure therapy in outpatient practice: a cluster randomized trial. J Am Coll Cardiol.

[51] Fuery MA, Kadhim B, Samsky MD, Freeman JV, Clark K, Desai NR (2023). Electronic health record embedded strategies for improving care of patients with heart failure. Curr Heart Fail Rep.

[52] McCarren M, Furmaga E, Jackevicius CA, Sahay A, Coppler TL, Katzianer J (2013). Improvement of guideline β-blocker prescribing in heart failure: a cluster-randomized pragmatic trial of a pharmacy intervention. J Card Fail.

[53] Mukhopadhyay A, Reynolds HR, Phillips LM, Nagler AR, King WC, Szerencsy A (2023). Cluster-randomized trial comparing ambulatory decision support tools to improve heart failure care. J Am Coll Cardiol.

[54] Ghazi L, Yamamoto Y, Fuery M, O’Connor K, Sen S, Samsky M, Riello RJ, Dhar R, Huang J, Olufade T, McDermott J, Inzucchi SE, Velazquez EJ, Wilson FP, Desai NR, Ahmad T. Electronic health record alerts for management of heart failure with reduced ejection fraction in hospitalized patients: the PROMPT-AHF trial. Eur Heart J. 2023;ehad512. 10.1093/eurheartj/ehad512 37650264

[55] Ahmad T, Desai NR, Yamamoto Y, Biswas A, Ghazi L, Martin M (2022). Alerting clinicians to 1-year mortality risk in patients hospitalized with heart failure: the REVEAL-HF randomized clinical trial. JAMA Cardiol.

[56] Man JP, Dijkgraaf MGW, Handoko ML, de Lange FJ, Winter MM, Schijven MP, Stienen S, Meregalli P, Kok WEM, Kuipers DI, van der Harst P, Koole MAC, Chamuleau SAJ, Schuuring MJ (2024). Digital consults to optimize guideline-directed therapy: design of a pragmatic multicenter randomized controlled trial. ESC Heart Fail.

[57] Gulizia MM, Orso F, Mortara A, Lucci D, Aspromonte N, De Luca L (2022). BLITZ-HF: a nationwide initiative to evaluate and improve adherence to acute and chronic heart failure guidelines. Eur J Heart Fail.

[58] Lynch KA, Ganz DA, Saliba D, Chang DS, de Peralta SS (2022). Improving heart failure care and guideline-directed medical therapy through proactive remote patient monitoring-home telehealth and pharmacy integration. BMJ Open Qual.

[59] Slade J, Lee M, Park J, Liu A, Heidenreich P, Allaudeen N (2022). Harnessing the potential of primary care pharmacists to improve heart failure management. Jt Comm J Qual Patient Saf.

[60] Rahimi K, Nazarzadeh M, Pinho-Gomes A-C, Woodward M, Salimi-Khorshidi G, Ohkuma T (2020). Home monitoring with technology-supported management in chronic heart failure: a randomised trial. Heart.

[61] Verma A, Fonarow GC, Hsu JJ, Jackevicius CA, Vaghaiwalla Mody F, Nguyen A (2023). DASH-HF study: a pragmatic quality improvement randomized implementation trial for patients with heart failure with reduced ejection fraction. Circ Heart Fail.

[62] Barsom EZ, Meijer HAW, Blom J, Schuuring MJ, Bemelman WA, Schijven MP (2021). Emergency upscaling of video consultation during the COVID-19 pandemic: contrasting user experience with data insights from the electronic health record in a large academic hospital. Int J Med Inform.

[63] Schuuring MJ, Kauw D, Bouma BJ (2020). COVID-19 pandemic: practical considerations on rapid initiation of remote care in chronic cardiac patients. Europ Heart J - Digital Health.

[64] Hrynyschyn R, Prediger C, Stock C, Helmer SM (2022). Evaluation methods applied to digital health interventions: what is being used beyond randomised controlled trials?-A scoping review. Int J Environ Res Public Health.

[65] Soobiah C, Cooper M, Kishimoto V, Bhatia RS, Scott T, Maloney S (2020). Identifying optimal frameworks to implement or evaluate digital health interventions: a scoping review protocol. BMJ Open.

[66] de Jong MJ, van der Meulen-de Jong AE, Romberg-Camps MJ, Becx MC, Maljaars JP, Cilissen M, van Bodegraven AA, Mahmmod N, Markus T, Hameeteman WM, Dijkstra G, Masclee AA, Boonen A, Winkens B, van Tubergen A, Jonkers DM, Pierik MJ (2017). Telemedicine for management of inflammatory bowel disease (myIBDcoach): a pragmatic, multicentre, randomised controlled trial. Lancet.

[67] Kauw D, Huisma PR, Medlock SK, Koole MAC, Wierda E, Abu-Hanna A, Schijven MP, Mulder BJM, Bouma BJ, Winter MM, Schuuring MJ (2020). Mobile health in cardiac patients: an overview on experiences and challenges of stakeholders involved in daily use and development. BMJ Innovations.

[68] Stevenson LW, Ross HJ, Rathman LD, Boehmer JP (2023). Remote monitoring for heart failure management at home. J Am Coll Cardiol.

[69] Schuuring MJ, Man JP, Chamuleau SAJ. Inclusive health tracking: unlock the true potential of digital health solutions. JACC Advances. 2023;2(7):100545. 10.1016/j.jacadv.2023.100545 10.1016/j.jacadv.2023.100545 PMC11198698 38939485

[70] Victoria-Castro AM, Martin M, Yamamoto Y, Ahmad T, Arora T, Calderon F (2022). Pragmatic randomized trial assessing the impact of digital health technology on quality of life in patients with heart failure: design, rationale and implementation. Clin Cardiol.

